# Correlations between glycosylated hemoglobin and glucose levels in Chinese older adults with newly diagnosed type 2 diabetes mellitus

**DOI:** 10.55730/1300-0144.5425

**Published:** 2022-05-07

**Authors:** Yun YU, Jun YANG, Wei TANG

**Affiliations:** Department of Endocrinology and Metabolism, Geriatric Hospital of Nanjing Medical University, Nanjing, China

**Keywords:** Glycosylated hemoglobin, older people, OGTT, prediabetes, type 2 diabetes mellitus

## Abstract

**Background/aim:**

To explore the correlations between glycosylated hemoglobin (HbA1c) and glucose levels in older adults with newly diagnosed type 2 diabetes mellitus (T2DM).

**Materials and methods:**

A total of 783 participants aged ≥60 years were enrolled. The 75-g oral glucose tolerance test (OGTT) was conducted and HbA1c was measured. The participants were divided into normal glucose tolerance (NGT)_HbA1c_, Pre-DM_HbA1c_, and T2DM_HbA1c_ groups based on the HbA1c diagnostic criteria. The correlations between HbA1c and glucose levels of the OGTT were analyzed.

**Results:**

When HbA1c ≥ 6.5% in older people, HbA1c was positively correlated with Glucose 0 min and 120 min of the OGTT (*r* = 0.335, 0.247; all p < 0.05, respectively). When HbA1c was between 5.7% and 6.4%, HbA1c was positively correlated with Glucose 0 min and 120 min (*r* = 0.298, 0.474; all p < 0.01, respectively). When HbA1c ≤ 5.6%, HbA1c was positively correlated with Glucose 0 min and 120 min (*r* = 0.301, 0.357; all p < 0.01, respectively). HbA1c was positively correlated with HOMA-IR (*r* = 0.368, p < 0.01), while it was negatively correlated with HOMA-β, ΔI30/ΔG30, IG120, and GDI(*r* = −0.267, −0.397,−0.364,−0.397; all p < 0.01, respectively). After adjustment for confounders, the correlations of HbA1c with Glucose 0 min and 120 min, insulin sensitivity and β-cell function indexes still existed. When HbA1c ≥ 6.5%, there were 93.3% T2DM_OGTT_ and 6.7% Pre-DM_OGTT_ subjects. When HbA1c < 6.5%, there were 17.7% T2DM_OGTT_, 39.5% Pre-DM_OGTT_ (including 2.5% IFG_OGTT_, 36.1% IGT_OGTT_ and 0.9% IGR_OGTT_), and 42.8% NGT_OGTT_ subjects.

**Conclusion:**

When HbA1c ≥ 6.5% in older people, HbA1c shows the highest correlation with Glucose 0 min of the OGTT. When HbA1c < 6.5%, postprandial hyperglycemia is a main characteristic of older people, and HbA1c shows the highest correlation with Glucose 120 min of the OGTT.

## 1. Introduction

The prevalence of type 2 diabetes mellitus (T2DM) increases dramatically especially in older people. A recent epidemiological survey from China reported that 28.8% of the older population (aged between 60 and 69 years) had diabetes and 31.8% met the diagnostic criteria for diabetes in the older population aged >70 years [1].

Older people are more likely to develop T2DM. The decreased muscle mass, increased visceral adiposity, and β-cell dysfunction with advancing age often lead to abnormal glucose metabolism [2]. Furthermore, some studies have presented that the main characteristic of glucose intolerance in older people is an increase in postprandial glucose, while fasting plasma glucose (FPG) levels are usually modestly elevated [3]. Epidemiologic data indicated that postprandial hyperglycemia and glucose fluctuation were the high risk factors of cardiovascular disease [4–6]. Thus, it is necessary to focus on the hyperglycemia in older people, especially postprandial hyperglycemia.

In 2010, the American Diabetes Association (ADA) recommended that either the 75-g oral glucose tolerance test (OGTT) or glycosylated hemoglobin (HbA1c) ≥ 6.5% could be used as diagnostic criteria for diabetes [7]. As we all know, HbA1c reflects the mean plasma glucose levels over the past 2 to 3 months and correlates with FPG or postprandial plasma glucose [8]. However, relative contributions of fasting and postprandial hyperglycemia to HbA1c had variations with increasing levels of HbA1c. When HbA1c was less than 7.3%, postprandial glucose contributed greater, accounting for about 70% [9]. Some of the older people had increased postprandial glucose but normal levels of FPG or HbA1c, but this issue was often ignored by clinicians. Therefore, although the detection of FPG or HbA1c is convenient, it is easy to miss the diagnosis of diabetes in older people even when combined with FPG and HbA1c [10]. This study was undertaken to investigate correlations between HbA1c and glucose levels in different HbA1c levels, and whether OGTT is required to diagnose diabetes when HbA1c is less than 6.5% in older Chinese people.

## 2. Methods

### 2.1. Subjects and research design

This cross-sectional study was conducted in our hospital from January 1 to December 31, 2020. A total of 783 subjects aged ≥60 years were consecutively recruited according to the set exclusion criteria: (i) previously diagnosed prediabetes (Pre-DM) or diabetes; (ii) active cancer, autoimmune disease, or infections and other inflammatory conditions; (iii) severe liver or renal impairment; (iv) a history of taking any medication known to affect glucose tolerance; and (v) clinical diagnosis of anemia or being on iron supplement at recruitment.

Glucose metabolism statuses were defined according to the American Diabetes Association (ADA) 2010 criteria [7]. According to HbA1c, glucose metabolism statuses were classified as follows: (i) normal glucose tolerance (NGT)_HbA1c_: HbA1c ≤ 5.6%; (ii) Pre-DM_HbA1c_: HbA1c between 5.7 and 6.4%; (iii) T2DM_HbA1c_: HbA1c ≥ 6.5%. Based on the OGTT, glucose metabolism statuses were classified as follows: (i) NGT_OGTT_: FPG < 5.6 mmol/L and 2h plasma glucose (2h PG) < 7.8 mmol/L; (ii) Pre-DM_OGTT_: impaired fasting glucose (IFG)_OGTT_: FPG between 5.6 and 6.9 mmol/L and 2h PG < 7.8 mmol/L; impaired glucose tolerance (IGT)_OGTT_: FPG < 5.6 mmol/L and 2h PG between 7.8 and 11.0 mmol/L; impaired glucose regulation (IGR)_OGTT_: FPG between 5.6 and 6.9 mmol/L and 2h PG between 7.8 and 11.0 mmol/L; (iii) T2DM_OGTT_: FPG ≥ 7.0 mmol/L and/or 2h PG ≥ 11.1 mmol/L.

Our study protocol was in accordance with Helsinki declaration and approved by the ethics committee of Geriatric Hospital of Nanjing Medical University. A written informed consent was obtained from each subject.

### 2.2. Anthropometric and biochemical measurements

Demographic data was collected from all subjects (age and sex), and all subjects underwent physical examination, including measurement of height, body weight, and waist circumference. Overnight fasting blood samples were obtained to test for hemoglobin, HbA1c, liver and renal functions, and lipid profiles. An OGTT was performed to collect blood samples at 0, 30, 60, and 120 min of the OGTT to measure glucose and insulin levels. Plasma glucose, liver and renal functions, and lipid profiles were measured using a Hitachi 7180 automated analyzer (Hitachi High-Tech Science Systems Corporation, Hitachinaka-shi, Japan). Hemoglobin was measured using a Hematology analyzer (Beckman Coulter Inc, California, USA). HbA1c was measured with a high-performance liquid chromatography analyzer (Bio-Rad Labs, Brea, CA, USA). Serum insulin was measured with radioimmunoassay (Roche Diagnostics, Mannheim, Germany).

### 2.3. Calculations

ΔI30/ΔG30, which reflects the early-phase β-cell function, was calculated as (insulin at 30 min of the OGTT–fasting insulin (FINS)) / ( glucose at 30 min of the OGTT–FPG) [11]. The area under the curve (AUC) of glucose and insulin during the OGTT was calculated with the trapezoidal method. GluAUC120 and InsAUC120 are the AUC of glucose and insulin during 0 to 120 min of the OGTT, respectively [12]. IG120 (InsAUC120/GluAUC120) was calculated as index adjusted for the corresponding glucose AUC [12] and reflects the total phase β-cell function. In addition, glucose disposition index (GDI) = ΔI30 / ΔG30 × (1 / FINS) [13].

Insulin resistance index (HOMA-IR) was calculated as FPG×FINS/22.5, and β-cell function (HOMA-β) was calculated as 20 × FINS /(FPG-3.5) [14].

### 2.4. Statistical analysis

Statistical analysis was performed with IBM SPSS (version 20.0, IBM Corp., Armonk, NY, USA). Continuous data were expressed as mean ± standard deviation (SD), and categorical variables were expressed as numbers (proportions). Since HOMA-IR and HOMA-β were abnormally distributed continuous variables, they were transformed by taking logarithm in order to be approximated by normal distribution before analysis. One-factor analysis of variance (ANOVA) was used to compare differences of clinical characteristics, insulin sensitivity, and β-cell function among different glucose metabolism statuses diagnosed by HbA1c. Chi-squared tests were performed on categorical variables. The correlations between HbA1c and glucose levels of OGTT, insulin sensitivity or β-cell function indexes were analyzed with Pearson’s correlation and further analyzed with partial correlation analysis adjusted for confounders such as age, sex, BMI, TG, and HDL-C. Student’s *t*-test was used to compare differences between IGT_OGTT_ and T2DM_OGTT_ both with HbA1c < 6.5%. ROC curve analysis was performed to determine the best cut-off levels of HbA1c for diagnosing Pre-DM and T2DM. The tests were performed at the significance level of p < 0.05.

## 3. Results

### 3.1. Comparison of clinical characteristics, insulin sensitivity and β-cell function among groups of different HbA1c levels

The total 783 subjects had a mean age of 69.68 ± 7.70 years, and with 480 males and 303 females. Based on the HbA1c diagnostic criteria, there were 455 NGT_HbA1c_ subjects, 224 Pre-DM_HbA1c_ patients and 104 T2DM_HbA1c_ patients.

The clinical characteristics, insulin sensitivity and β-cell function among different levels of HbA1c are described in Table 1. Notably, there were statistically significant increasing trends in BMI, WC, HbA1c, glucose levels at 0, 30, 60, or 120 min of the OGTT, TG (triglycerides), and HOMA-IR among the different HbA1c groups; furthermore, there were decreasing trends in high-density lipoprotein cholesterol (HDL-C), HOMA-β, ΔI30/ΔG30, IG120, and GDI (all p < 0.05). There was no significant difference in age, sex, hemoglobin, total cholesterol (TC), low-density lipoprotein cholesterol (LDL-C), and liver and renal functions among the different HbA1c groups (all p > 0.05).

### 3.2. Distribution of NGT_OGTT_, Pre-DM_OGTT_, and T2DM_OGTT_ subjects among groups of different HbA1c levels

As shown in Table 2, 104 T2DM_HbA1c_ patients included 97 (93.3%) T2DM_OGTT_ and 7 (6.7%) Pre-DM_OGTT_ patients. Furthermore, in 224 Pre-DM_HbA1c_ subjects, there were 16 (7.1%) NGT_OGTT_ subjects, 108 (48.2%) Pre-DM_OGTT_ patients (92.6% were IGT_OGTT_ patients), and 100 (44.7%) T2DM_OGTT_ subjects (84.0% were diagnosed with diabetes by Glucose 120 min of the OGTT alone). Additionally, in 455 NGT_HbA1c_ subjects, 275 (60.4%) were NGT_OGTT_ subjects, 160 (35.2%) were Pre-DM_OGTT_ patients (90.6% were IGT_OGTT_ patients), and even 20 (4.4%) were T2DM_OGTT_ (100% were all diagnosed with diabetes by Glucose 120 min of the OGTT alone).

### 3.3. Correlations of HbA1c with glucose levels of OGTT, insulin sensitivity, and β-cell function indexes

Correlations between HbA1c and glucose levels of OGTT at different levels of HbA1c are presented in Table 3. When HbA1c ≥ 6.5% in older people, HbA1c was positively correlated with Glucose 0 min, 30 min, 60 min,120 min of the OGTT (*r* = 0.335, 0.232, 0.223, 0.247; all p < 0.05, respectively). When HbA1c was between 5.7% and 6.4%, HbA1c was positively correlated with Glucose 0 min, 30 min, 60 min, 120 min (*r* = 0.298, 0.206, 0.382, 0.474; all p < 0.05, respectively). When HbA1c ≤ 5.6%, HbA1c was positively correlated with Glucose 0 min, 30 min, 60 min,120 min (*r* = 0.301, 0.318, 0.280, 0.357 ; all p < 0.01, respectively). We further analyzed the correlation between HbA1c and insulin sensitivity and **β-cell** function indexes. As presented in Table 4, HbA1c was positively correlated with HOMA-IR (*r* = 0.368, p < 0.01), while it was negatively correlated with HOMA-β, ΔI30/ΔG30, IG120, and GDI (*r* = −0.267, −0.397, −0.364, −0.397; all p < 0.01, respectively).

After adjustment for confounders such as age, sex, BMI, TG, and HDL-C, the correlations of HbA1c with Glucose 0 min and 120 min of the OGTT, insulin sensitivity, and β-cell function indexes, the correlations mentioned-above still existed.

### 3.4. Comparison of clinical characteristics, insulin sensitivity and β-cell function between IGT_OGTT_ and T2DM_OGTT_ both with HbA1c < 6.5%

In 679 subjects all with HbA1c levels < 6.5%, there were 245 (36.1%) IGT_OGTT_ and 120 (17.7%) T2DM_OGTT_ subjects. HbA1c levels in the IGT_OGTT_ group were between 4.6% and 6.4%, with an average of 5.81 ± 0.38%; HbA1c levels in the T2DM_OGTT_ group were between 5.4% and 6.4%, with an average of 6.13 ± 0.25%, and the mean levels of HbA1c in the IGT_OGTT_ were significantly lower than those of the T2DM_OGTT_ groups (p < 0.01). As shown in Table 5, HOMA-IR in the IGT_OGTT_ group was significantly lower than that in the T2DM_OGTT_ group (p < 0.01), whileΔI30/ΔG30, IG120, and GDI in the IGT_OGTT_ group were significantly higher than those in the T2DM_OGTT_ group (all p < 0.05). However, HOMA-β showed no significant differences between two groups (p > 0.05). Additionally, there were no significant differences in age, sex, BMI, and hemoglobin between the two groups (all p > 0.05).

### 3.5. ROC curves for the best cut-off levels of HbA1c for diagnosing Pre-DM and T2DM

**Figure 1:**
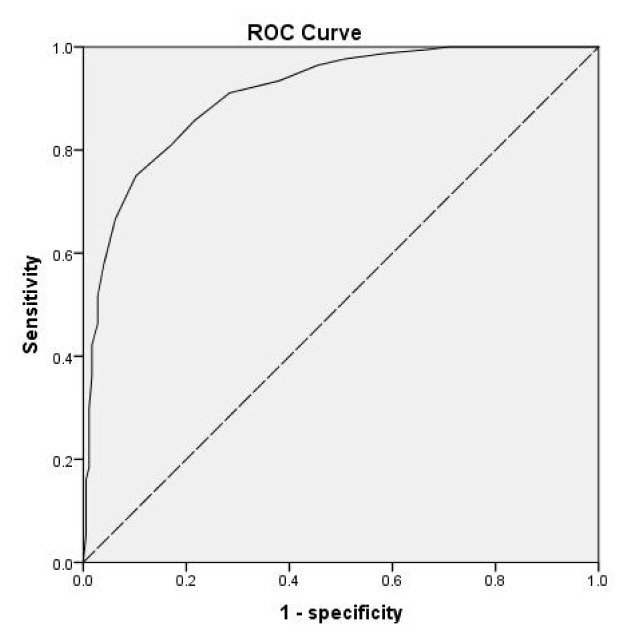
The ROC curve for diabetes diagnosis using HbA1c, with OGTT as the reference standard.

**Figure 2:**
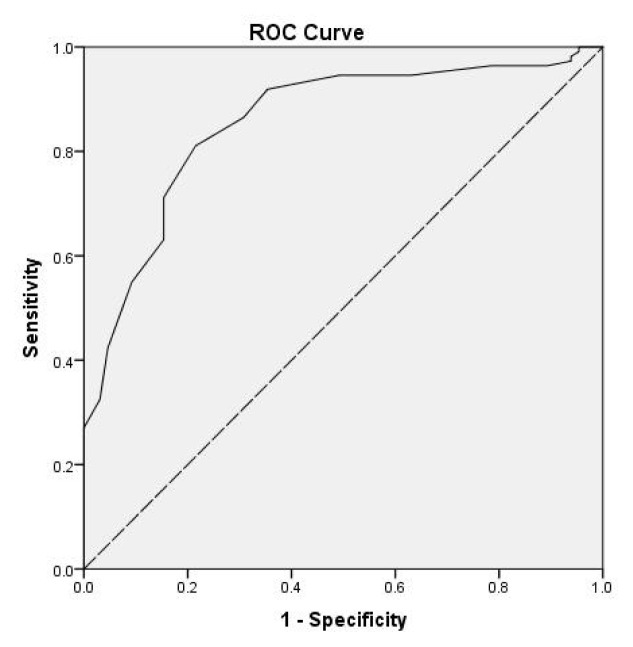
The ROC curve for Pre-DM diagnosis using HbA1c, with OGTT as the reference standard.

In our study population, the best HbA1c cut-off value for diagnosis of T2DM was 6.25% (AUC 0.909, 95% CI 0.879–0.939, p < 0.001), with a sensitivity of 75% and a specificity of 89.8% (Figure 1). Additionally, the cut-off value of HbA1c for diagnosing Pre-DM was 5.35% (AUC 0.856, 95% CI 0.798–0.913, p < 0.001), and the sensitivity was 91.9% and specificity was 64.6% (Figure 2).

## 4. Discussion

The present study showed that HbA1c showed the highest correlation with FPG when HbA1c ≥ 6.5% in the older people. When HbA1c was less than 6.5%, postprandial hyperglycemia was a main characteristic of the older people, and HbA1c showed the highest correlation with 2h PG. It was possible to diagnose diabetes with HbA1c ≥ 6.5%, while the OGTT was needed for diagnosing diabetes with HbA1c levels < 6.5% in the older people.

In 2010, the ADA recommended that either the OGTT or HbA1c ≥ 6.5% could be used as diagnostic criteria for diabetes [7]. Meanwhile, the WHO also adopted HbA1c as an alternative diagnostic method for diabetes in 2011 [15]. However, it remains controversial whether the diagnostic sensitivity of HbA1c to detect diabetes was high or not. One study showed that the HbA1c threshold of 6.5% had a good specificity and sensitivity for diagnosing diabetes [16]. However, other studies showed that the HbA1c test just had moderate sensitivity for diabetic diagnosis. Zhou et al. [17] reported that less than 30% of the newly diagnosed diabetes could be identified at the HbA1c cut-off point of ≥6.5%. Peter et al. [18] found that 29 % of the diabetic patients showed normal HbA1c levels (4.0~6.0%), and only 47% of the diabetic individuals were diagnosed correctly by the 6.5% cut-off value of HbA1c. Among the remaining 53% diabetic individuals with HbA1c < 6.5%, 35% had increased FPG values and 65% had increased 2h PG levels.

Glucose intolerance is associated with aging [19]. Previous studies reported that with age advancing, the 2h PG of an OGTT rose more steeply than FPG [20,21]. Most of the asymptomatic older patients with diabetes only showed postprandial hyperglycemia [22]. Therefore, diagnosis of diabetes could be made several years earlier using OGTT versus FPG alone in older people [23]. Our study found that in T2DM_HbA1c_ patients all with HbA1c ≥ 6.5%, patients with T2DM_OGTT_ accounted for 93.3%; while when HbA1c < 6.5%, there were 17.7% patients with T2DM_OGTT_, and 86.7% of the T2DM_OGTT_ patients were diagnosed with diabetes by Glucose 120 min of the OGTT alone. These results suggested that postprandial hyperglycemia was the main manifestation of older patients at lower HbA1c levels, and even if HbA1c was 5.7%~6.4% in the older adults, the possibility of having diabetes could not be excluded, and further OGTT was needed.

That HbA1c reflects contributions from both fasting and postprandial hyperglycemia is well understood [24]. In 2003, Monnier et al. [9] reported a landmark study describing the relative contributions of fasting and postprandial hyperglycemia at different levels of HbA1c. The findings suggested that the relative contribution of postprandial hyperglycemia decreased gradually while the relative contribution of fasting hyperglycemia increased progressively with increasing levels of HbA1c. These results also reflected a basic biological characteristic of T2DM: the postprandial hyperglycemia seemed to appear early in the natural history of T2DM, whereas the fasting hyperglycemia appeared later after further β-cell dysfunction [25]. Other studies also supported the relative contribution of postprandial hyperglycemia at lower HbA1c levels and fasting hyperglycemia at higher levels [26,27]. Our study also found that in subgroup of HbA1c ≤ 5.6% or HbA1c 5.7%~6.4%, HbA1c showed the highest correlation with 2hPG; however, in subgroup of HbA1c ≥ 6.5%, HbA1c showed the highest correlation with FPG in older adults.

It seems that OGTT and HbA1c reflect different physiopathological aspects of dysglycemia [28]. High 2hPG levels of the OGTT might show the inability of handling an acute glucose load characterized by changes in insulin secretion and action, while HbA1c may represent mean levels of glycaemic alteration [29]. In this study, we found that in 679 older subjects all with HbA1c < 6.5%, there were 36.1% IGT_OGTT_ patients and 17.7% T2DM_OGTT_ patients, and the mean levels of HbA1c in the IGT_OGTT_ were significantly lower than those in the T2DM_OGTT_ groups. The differences of insulin sensitivity and β-cell function were further compared between the two groups. We found that the levels of HOMA-IR in patients with IGT_OGTT_ were significantly lower while the levels of β-cell function indexes (I30/ΔG30, IG120) and GDI were significantly higher than those with T2DM_OGTT_. The results showed that the IGT_OGTT_ group had milder insulin resistance, relatively better early-phase insulin secretion, total insulin secretion, and compensatory ability of β-cells compared with those of the T2DM_OGTT_ group even though HbA1c levels were both less than 6.5%. It was further suggested that OGTT should be performed in older people with HbA1c < 6.5% to clarify the state of glucose metabolism.

The limitations of our study are as follows. Firstly, the study was cross-sectional, it had a small sample size, and it lacked diabetic complications data. Therefore, further large-scale and prospective studies are warranted. In addition, we used the homeostasis model assessment (HOMA) and other indexes to assess insulin sensitivity and β-cell function instead of the hyperglycemic clamp technique, which may cause biases.

## 5. Conclusion

When HbA1c ≥ 6.5% in older Chinese adults, HbA1c showed the highest correlation with FPG. When HbA1c was less than 6.5%, HbA1c showed the highest correlation with 2hPG, and the possibility of having diabetes could not be excluded, and further OGTT was needed.

## Figures and Tables

**Table 1:** Comparison of clinical characteristics, insulin sensitivity and β-cell function among different levels of HbA1c.

Parameters assessed	NGT_HbA1c_	Pre-DM_HbA1c_	T2DM_HbA1c_	p-value‡
n(male/female)†	455(270/185)	224(140/84)	104(70/34)	0.503
Age (years)	69.10 ± 6.33	70.49 ± 8.13	69.03 ± 8.11	0.234
BMI (kg/m^2^)	24.20 ± 3.21	25.59 ± 2.94**	26.61 ± 4.17**Δ	<0.001
WC (cm)	84.49 ± 9.93	90.40 ± 9.18**	92.12 ± 10.77**	<0.001
TG (mmol/L)	1.69 ± 1.14	1.87 ± 0.90*	2.06 ± 1.20*ΔΔ	0.001
TC (mmol/L)	4.72 ± 0.98	4.72 ± 0.98	4.63 ± 0.99	0.754
LDL-C(mmol/L)	2.62 ± 0.79	2.80 ± 0.83	2.74 ± 0.79	0.284
HDL-C(mmol/L)	1.23 ± 0.37	1.15 ± 0.31*	1.07 ± 0.30**Δ	0.005
Hemoglobin (g/L)	134.73 ± 14.57	133.24 ± 14.26	136.74 ± 16.16	0.195
HbA1c (%)	5.26 ± 0.31	6.07 ± 0.22**	6.98 ± 0.32**ΔΔ	<0.001
ALT (U/L)	25.10 ± 11.99	25.24 ± 13.90	26.70 ± 15.74	0.728
AST (U/L)	23.71 ± 8.50	23.50 ± 13.02	23.99 ± 11.74	0.958
BUN (mmol/L)	5.57 ± 1.19	5.76 ± 1.31	5.56 ± 1.47	0.461
SCr (μmol/L)	74.39 ± 15.08	71.77 ± 19.38	71.82 ± 17.00	0.614
Glucose 0 min (mmol/L)	5.12 ± 0.59	5.73 ± 0.85**	6.79 ± 1.17**ΔΔ	<0.001
Glucose 30 min (mmol/L)	9.10 ± 1.79	10.73 ± 1.70**	12.38 ± 1.82**ΔΔ	<0.001
Glucose 60 min (mmol/L)	9.17 ± 2.40	12.26 ± 2.95**	15.26 ± 2.41**ΔΔ	<0.001
Glucose120 min (mmol/L)	6.90 ± 2.16	10.77 ± 3.22**	14.80 ± 2.94**ΔΔ	<0.001
HOMA-IR	0.50 ± 0.56	0.89 ± 0.63**	1.10 ± 0.64**ΔΔ	<0.001
HOMA-β	4.57 ± 0.65	4.34 ± 0.60*	4.18 ± 0.54**ΔΔ	<0.001
ΔI30/ΔG30	19.39 ± 11.29	11.10 ± 9.51**	5.37 ± 3.47**ΔΔ	<0.001
IG120	7.76 ± 4.53	6.64 ± 4.12*	4.00 ± 2.59**ΔΔ	<0.001
GDI	2.75 ± 1.03	1.15 ± 0.97**	0.53 ± 0.34**Δ	<0.001

Data are presented as mean ± SD.

HOMA-IR and HOMA-β were Ln-transformed because of abnormal distribution.

† Number of people.

‡ p-value was the overall comparison of three different HbA1c level groups.

* p < 0.05 compared with the NGT_HbA1c_ group,

** p < 0.01 compared with the NGT_HbA1c_ group.

Δ p < 0.05 compared with the Pre-DM_HbA1c_ group,

ΔΔ p < 0.01 compared with the Pre-DM_HbA1c_ group.

Abbreviations: BMI, body mass index; WC, waist circumference; SBP, systolic blood pressure; DBP, diastolic blood pressure; TG, triglyceride; TC, total cholesterol; LDL-C, low-density lipoprotein cholesterol; HDL-C, high-density lipoprotein cholesterol; HbA1c, glycosylated hemoglobin; ALT, alanine aminotransferase; AST, aspartate aminotransferase; BUN, blood urea nitrogen; SCr, serum creatinine; ΔI30/ΔG30, (insulin at 30 min of the OGTT–fasting insulin)/(glucose at 30 min of the OGTT–fasting glucose); IG120, InsAUC120/GluAUC120(area under the curve of glucose from 0 to 120 min of the OGTT); GDI, glucose disposition index.

**Table 2:** Distribution of subjects with NGT_OGTT_, Pre-DM_OGTT_, and T2DM_OGTT_ among different levels of HbA1c.

OGTT	NGT_HbA1c_ (n = 455)	Pre-DM_HbA1c_ (n = 224)	T2DM_HbA1c_ (n = 104)
NGT_OGTT_	275 (60.4%)	16 (7.1%)	0 (0.0%)
Pre-DM_OGTT_	160 (35.2%)	108 (48.2%)	7 (6.7%)
IFG_OGTT_	15 (3.3%)	2 (0.8%)	0 (0.0%)
IGT_OGTT_	145 (31.9%)	100 (44.7%)	4 (3.8%)
IGR_OGTT_	0 (0.0%)	6 (2.7%)	3 (2.9%)
T2DM_OGTT_	20 (4.4%)	100 (44.7%)	97 (93.3%)
Diagnosed by FPG alone	0 (0.0)	3 (1.3)	2 (1.9)
Diagnosed by 2h PG alone	20 (4.4)	84 (37.5)	55 (52.9)
Diagnosed by FPG +2h PG	0 (0.0)	13 (5.9)	40 (38.5)

Values are expressed as numbers (percentages).

**Table 3:** Correlations between HbA1c and glucose levels of OGTT among different levels of HbA1c.

OGTT	HbA1c ≤ 5.6%				HbA1c 5.7%~6.4%				HbA1c ≥ 6.5%			
	Crude		Multiple adjusted†		Crude		Multiple adjusted†		Crude		Multiple adjusted†	
	*r*	p-value	*r*	p-value	*r*	p-value	*r*	p-value	*r*	p-value	*r*	p-value
Glucose 0 min	0.301	0.004	0.289	0.009	0.298	<0.001	0.294	0.001	0.335	0.001	0.308	0.002
Glucose 30 min	0.318	0.002	0.291	0.016	0.206	0.012	0.223	0.011	0.232	0.018	0.148	0.147
Glucose 60 min	0.280	0.007	0.231	0.058	0.382	<0.001	0.409	< 0.001	0.223	0.023	0.146	0.153
Glucose 120 min	0.357	0.001	0.328	0.006	0.474	<0.001	0.493	< 0.001	0.247	0.011	0.242	0.017

† Multiple adjusted for variables including: age (years), sex, BMI (kg/m^2^), TG (mmol/L), and HDL-C (mmol/L).

**Table 4:** Correlations between HbA1c and insulin sensitivity or β-cell function indexes.

	HbA1c					
	Crude		Adjusted for age, sex		Multiple adjusted†	
	*r*	p-value	*r*	p-value	*r*	p-value
HOMA-IR	0.368	<0.001	0.362	<0.001	0.237	<0.001
HOMA-β	−0.267	<0.001	−0.272	<0.001	−0.348	<0.001
**ΔI30/ΔG30**	−0.397	<0.001	−0.394	<0.001	−0.413	<0.001
IG120	−0.364	<0.001	−0.395	<0.001	−0.425	<0.001
GDI	−0.397	<0.001	−0.362	<0.001	−0.401	<0.001

† Multiple adjusted for variables including: age (years), sex, BMI (kg/m^2^), TG (mmol/L), and HDL-C (mmol/L).

**Table 5:** Comparison of clinical characteristics, insulin sensitivity and β-cell function between IGT_OGTT_ and T2DM_OGTT_ both with HbA1c < 6.5%.

Parameters assessed	IGT_OGTT_	T2DM_OGTT_	p-value‡
n(male/female) †	245 (140/105)	120 (71/49)	0.164
Age (years)	71.24 ± 7.99	69.35 ± 7.96	0.133
BMI (kg/m^2^)	25.29 ± 3.19	25.76 ± 3.14	0.345
WC (cm)	88.59 ± 10.72	91.93 ± 8.49	0.057
Hemoglobin (g/L)	133.54 ± 15.54	135.95 ± 15.41	0.423
HbA1c (%)	5.81 ± 0.38	6.13 ± 0.25	<0.001
Glucose 0 min (mmol/L)	5.42 ± 0.56	6.06 ± 0.91	<0.001
Glucose 30 min (mmol/L)	10.15 ± 1.37	11.48 ± 1.60	<0.001
Glucose 60 min (mmol/L)	11.01 ± 2.17	13.89 ± 2.52	<0.001
Glucose120 min (mmol/L)	8.76 ± 1.48	13.49 ± 2.17	<0.001
Insulin 0 min (uIU/L)	10.03 ± 6.66	12.71 ± 6.80	0.012
Insulin 30min (uIU/L)	67.43 ± 45.02	59.79 ± 42.88	0.270
Insulin 60 min (uIU/L)	86.44 ± 58.75	80.06 ± 57.01	0.484
Insulin 120 min (uIU/L)	79.52 ± 55.70	100.00 ± 58.70	0.023
HOMA-IR	0.71 ± 0.61	1.08 ± 0.60	<0.001
HOMA-β	4.53 ± 0.61	4.54 ± 0.61	0.936
ΔI30/ΔG30	13.31 ± 11.05	8.61 ± 5.92	0.001
IG120	7.38 ± 4.43	5.99 ± 3.69	0.033
GDI	1.46 ± 0.99	0.74 ± 0.42	<0.001

Data are presented as mean ± SD.

HOMA-IR and HOMA-β were Ln-transformed because of abnormal distribution.

† Number of people.

‡ p-value was the comparison between the IGT_OGTT_ and T2DM_OGTT_ groups.

Abbreviations: BMI, body mass index; WC, waist circumference; HbA1c, glycosylated hemoglobin; ΔI30/ΔG30, (insulin at 30 min of the OGTT–fasting insulin)/( glucose at 30 min of the OGTT–fasting glucose); IG120, InsAUC120/GluAUC120 (area under the curve of glucose from 0 to 120 min of the OGTT); GDI, glucose disposition index.
